# The Prognostic and Clinical Value of Tumor-Associated Macrophages in Patients With Breast Cancer: A Systematic Review and Meta-Analysis

**DOI:** 10.3389/fonc.2022.905846

**Published:** 2022-06-30

**Authors:** Changjun Wang, Yan Lin, Hanjiang Zhu, Yidong Zhou, Feng Mao, Xin Huang, Qiang Sun, Chenggang Li

**Affiliations:** ^1^ Department of Breast Surgery, Peking Union Medical College Hospital, Beijing, China; ^2^ Department of Dermatology, 90 Medical Center Way, Surge 110, University of California, San Francisco, San Francisco, CA, United States; ^3^ State Key Laboratory of Medicinal Chemical biology, Nankai University, Tianjin, China; ^4^ College of Pharmacy, Nankai University, Tianjin, China

**Keywords:** tumor-associated macrophages, breast cancer, survival, systematic review, meta-analysis

## Abstract

**Background:**

The prognostic and clinical value of tumor-associated macrophages (TAMs) in patients with breast cancer (BCa) remains unclear. We conducted the current meta-analysis to systematically evaluate the association of CD68+ and CD163+ TAM density with the prognosis and clinicopathologic features of BCa patients.

**Methods:**

Searches of Web of Science, PubMed, and EMBASE databases were performed up to January 31, 2022. The meta-analysis was conducted using hazard risks (HRs) and 95% confidence intervals (CIs) for survival data including overall survival (OS), disease-free survival (DFS), and BCa specific survival. Sensitivity and meta-regression analyses were also conducted to identify the robustness of the pooled estimates.

**Results:**

Our literature search identified relevant articles involving a total of 8,496 patients from 32 included studies. Our analysis indicates that a high CD68+ TAM density in the tumor stoma was significantly linked with poor OS (HR 2.46, 95% CI, 1.83–3.31, *P*<0.001) and shorter DFS (HR 1.77, 95% CI, 1.08–2.89, *P*=0.02) compared to low CD68+ TAM density. A significant association was also found in the tumor nest. Analysis of CD163+ TAM density showed similar results (all *P*<0.001). Notably, the pooled analysis with multivariate-adjusted HRs for OS and DFS also found that a high TAM density was significantly related to poorer outcomes for BCa patients (all *P*<0.05). In addition, BCa patients with high TAM density were more likely to have larger tumors, no vascular invasion, and positive estrogen receptor expression (all *P*<0.05).

**Conclusion:**

This meta-analysis indicates that a high CD68+ and CD163+ TAM density is associated with poor OS and shorter DFS in BCa patients. Further clinical studies and *in vivo* experiments are needed to elucidate the underlying mechanism of TAMs.

**Systematic Review Registration:**

https://www.crd.york.ac.uk/prospero/display_record.php?ID=CRD42022304853, identifier CRD42022304853.

## Introduction

Breast cancer (BCa) is one of the most frequent cancers among malignant diseases in women and is the leading cause of cancer-related deaths worldwide (1). Recently, BCa has exhibited a trend of early age onset, further threatening women’s health and global disease burden (2). Despite great achievements in the diagnosis and clinical treatment of BCa, overall survival (OS) has not significantly improved, especially for patients with advanced-stage or triple-negative BCa (3, 4). Traditional prognostic indicators, such as TNM classification scheme, histological grade, progesterone receptor (PR), estrogen receptor (ER), and human epidermal growth factor receptor-2 (HER2), can not fully represent tumor biological behavior and BCa prognosis (5–7). Therefore, there remains a large unmet demand for novel effective biomarkers with superior prognostic and predictive power to deliver personalized and precise treatment for BCa.

Recently, the tumor microenvironment (TME) has gained increased interest in BCa research. Both clinical and pre-clinical studies found a mixture of tumor cells and host-activated immune cells including B cells, natural killer cells, and tumor-associated macrophages (TAMs) that predominated on the BCa TME (8, 9). It was demonstrated that tumor-associated immune cells are associated with tumor progression, metastasis, and acquired resistance. TAMs are the main component of the TME, accounting for approximately 50% of TME cells, playing a crucial role in antigen presentation, angiogenesis, tissue repair, and tumor cell apoptosis (10). TAMs can be classified into two main functional subtypes including classically activated M1 and alternatively activated M2 macrophages (11). Generally, M1 macrophages exert cytotoxic effects on cancer cells *via* proinflammatory cytokine molecules such as lipopolysaccharide, interleukin-12, and interferon-γ. In contrast, M2 macrophages function as “tumor promotors”, which facilitate tumor cell invasion and metastasis and restrain anti-tumor immune response (9, 12).

Several studies focused on the prognostic significance of TAMs among different cancers, such as lung (13), liver (14), gastric (15), pancreatic (16) cancer, and BCa (17). The prognostic value of TAMs remains controversial and the results highly depend on macrophage subtypes and TAMs locations (18). This systematic review and meta‐analysis was conducted to evaluate the impact of different TAMs markers and histologic locations on BCa prognosis. We also analyzed the association between TAMs infiltration and BCa clinicopathologic features. A clearer understanding of TAMs infiltration modes and prognostic value would be helpful to improve treatment efficacy in BCa.

## Methods

This meta-analysis was performed in accordance with the Meta‐Analyses and Systematic Reviews of Observational Studies (MOOSE) (19) and Preferred Reporting Items for Systematic Reviews and Meta‐Analyses (PRISMA) guidelines (20). The meta-analysis is registered with PROSPERO (CRD42022304853).

### Literature Search

Two investigators (WCJ and LY) independently searched the Web of Science, PubMed (MEDLINE), and EMBASE databases for potential studies published in journals until January 31, 2022, without any language limitation. The main key words were “tumor-associated macrophages” + “breast cancer”, and a detailed search strategy is shown in **Supplementary Table 1**. We also conducted forward and backward citation tracking to avoid missing any relevant literature. Unpublished literature and conference papers were not included. All studies reporting TAMs and BCa were included and screened by two authors independently based on the inclusion criteria.

### Inclusion Criteria

We included studies reporting TAMs associated with BCa that met the following inclusion criteria: (i) patients with pathologically diagnosed BCa; (ii) BCa patients without any previous cancer history; (iii) TAMs were measured at the primary tumor site using immunohistochemistry (IHC) staining for CD68 and CD163; and (iv) the study design was a cohort study or case-control study, evaluating the association of TAMs with survival data [OS, breast cancer specific survival (BCSS), disease-free survival (DFS)] and other clinical outcomes.

### Exclusion Criteria

We excluded studies measuring TAMs at metastases or local relapse sites. Comments, reviews, conference abstracts, and case reports were also excluded from our meta-analysis.

### Quality Assessment and Data Extraction

The quality of each selected study was independently evaluated by two experienced researchers using the modified Newcastle–Ottawa Scale (NOS) based on the current PRISMA guidelines (21). The researchers focused on measurement and selection bias because most studies included in this review were cross-sectionally designed. Studies obtained a NOS score based on three evaluation indicators including study comparability, patient selection, and outcome assessment. Eligible studies were graded as high quality with a NOS score ≥6. A third researcher resolved any disagreements and made the final decision for candidate articles.

Two authors independently extracted the data from the studies using a standardized data extraction form. The following data were extracted: name of the first author, publication year, country, study design, study period, sample size, age, treatment received, tumor size, histologic type, histological grade, the status of ER, PR, HER-2, and Ki-67 (positive or negative), macrophage markers, macrophage location site [tumor nest (TN) or tumor stroma (TS)], follow-up time, OS, DFS, and BCSS with adjusted or unadjusted hazard ratios (HRs) and 95% confidence interval (CIs). TAMs in the TN was defined as intraepithelial tumor-infiltrating macrophages, and TS was defined as the stromal tissue surrounding the tumor nest. We also collected prognostic information from studies that only reported a Kaplan–Meier (KM) plot and a *P*-value derived from log-rank analysis. HRs and 95% CIs were extracted from KM plots using Engauge Digitizer version 4.1 (free software downloaded from http://sourceforge.net) and calculated as previously described (22) . The low TAM group was used as a reference to calculate HRs. If the high TAM group was considered as a reference in the included study, then the relevant measures were inverted to ensure data uniformity. The corresponding author of the included study was contacted if there were any unclear or missing data.

### Statistical Analysis

The statistical analysis was performed according to the recommendations from The Cochrane Collaboration. The HR with 95% CI was used to evaluate the association between TAM density and survival. The odds risk (OR) with 95% CI for the difference in clinicopathological features was used to measure dichotomous data. Heterogeneity across studies was assessed using the Cochran Q test and the *I^2^* statistics. For *I^2^* statistics, we considered *I^2^* <25% as low heterogeneity and *I^2^* > 5% as high heterogeneity. Data were also analyzed with a fixed-effects model for *P* > 0.10 and *I^2^* <50%; otherwise, the random-effects model was applied. We performed meta-regression analysis to analyze the role of potential contributors to heterogeneity using the “metafor” package in R software (Version 4.0.2; R Foundation for Statistical Computing, Vienna, Austria). Subgroup analysis and sensitivity analysis were also conducted to identify the source of heterogeneity. Potential publication bias was evaluated using funnel plots. All statistical analyses were conducted using Review Manager Version 5.3 software (The Nordic Cochrane Center, The Cochrane Collaboration, 2014, Copenhagen). A two-tailed *P*-value <0.05 was considered statistically significant.

## Results

**Figure 1:**
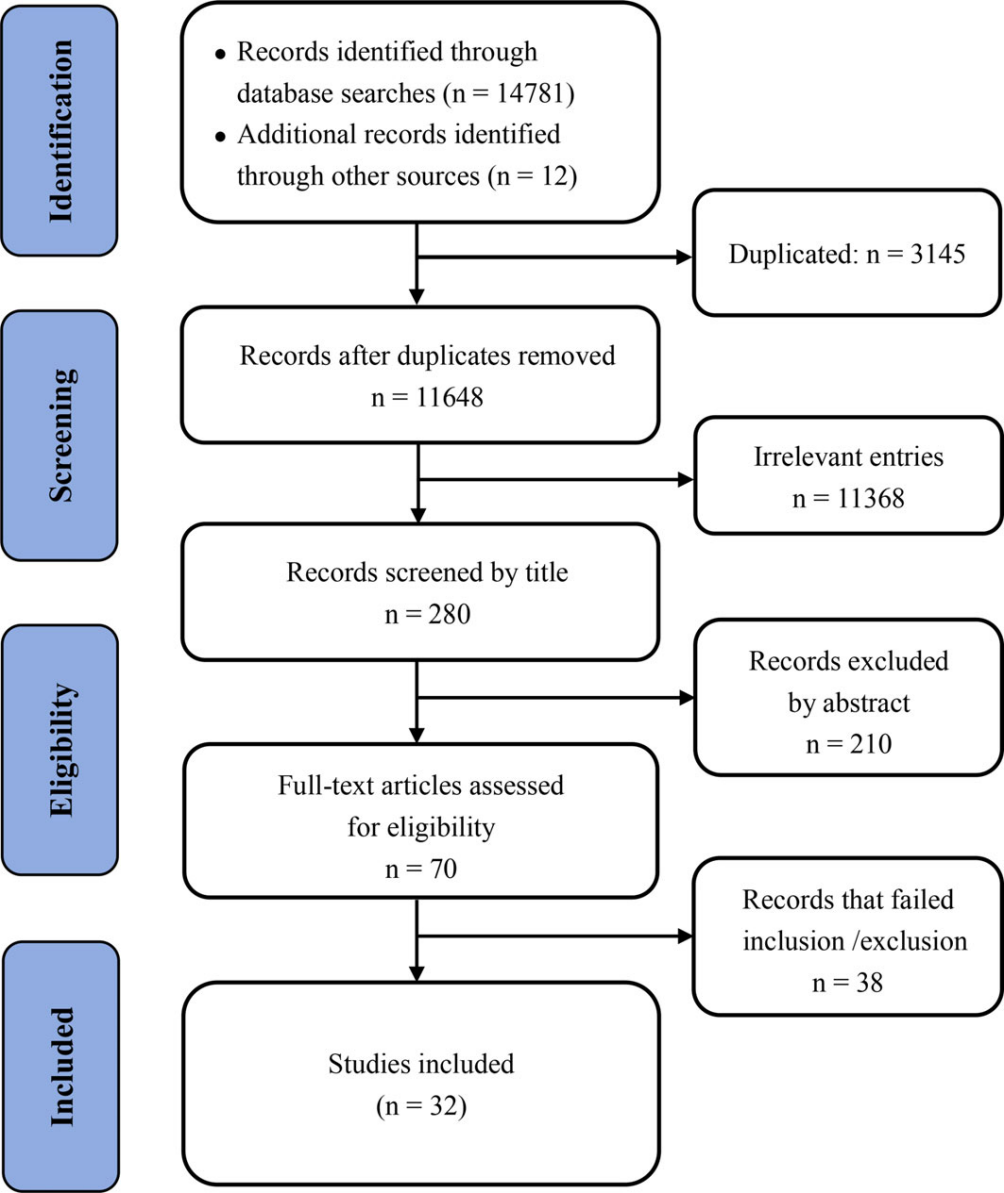
Flow diagram of article selection.

A total of 14,781 articles were found in our initial search, and 3,145 duplicated articles and irrelevant studies were removed. After reviewing the title and abstract, 11,368 studies were excluded; after reviewing the full text 38 articles were excluded. Finally, 32 unique studies were included in the meta-analysis (**Supplementary Table 2**). The detailed screening method and results are presented in **Figure 1**.

### Basic Characteristics and Quality Assessment

The main characteristics of the enrolled studies are summarized in **Table 1**. We included 32 studies in our meta-analysis that were published between 1996 and 2021 and conducted in 10 countries from 1985 to 2018 (England, Japan, America, UK, Sweden, China, Finland, Republic of Korea, Singapore, Germany). A total of 8,496 patients were included in the eligible studies, with the reported age from 23 to 97 years.

**Table 1:** Characteristics of studies included in the meta-analysis.

Author	Country	Sample size	Markers	Cut-off value	Tissue distribution	Analysis	Follow-up	Outcome assessment	Selection	Comparability	Outcome	NOS
Leek et al., 1996 (23)	England	91	CD68+	Median 12	Tumor nest	Unavailable	60 months	OS, DFS	★★★	★★	★	6
Tsutsui et al., 2005 (24)	Japan	249	CD68+	55th percentile	Tumor nest	Unavailable	Unavailable	DFS	★★★★	★★	★	7
Murri et al., 2008 (25)	UK	168	CD68+	Tertiles	Tumor nest	Blind	Median 72 months	OS, BCSS	★★★★	★	★★	7
Campbell et al., 2010 (26)	American	216	CD68+/ PCNA+	5	Tumor nest	Blind	108 months	OS, DFS	★★★	★★	★★★	8
Mukhtar et al., 2011 (27)	American	70	CD68+/ PCNA+	Median 5	Tumor nest	Blind	Median 10.34 years	OS, DFS	★★★	★★	★★★	8
Mohammed et al., 2012 (28)	UK	468	CD68+	Tertiles	Tumor nest	Blind	10 years	OS, BCSS	★★★★	★	★★★	8
Medrek et al. 2012 (29)	Sweden	144	CD68+ CD163+	Median 50%	Tumor nest and stroma	Unavailable	Median 6.55 years (0.33-7.55)	OS, BCSS, DFS	★★★★	★	★★★	8
Mahmoud et al. 2012 (30)	UK	1902	CD68+	TN, 6 TS,17	Tumor nest and stroma	Blind	Unavailable	OS, BCSS, DFS	★★★	★	★★	6
Carrio et al., 2012 (31)	American	29	CD68+	Positive	Tumor nest	Unavailable	Unavailable	OS	★★★	★	★★★	7
Zhang et al., 2013 (32)	China	172	CD68+	Median 26	Tumor nest	Blind	Unavailable	OS, DFS	★★★	★★	★★	7
Campbell et al., 2013 (33)	American	102	CD68+/PCNA+	Mean 24	Tumor nest	Unavailable	Unavailable	OS, DFS	★★★	★★	★★	7
Yuan et al., 2014 (34)	China	287	CD68+	16	Tumor stroma	Unavailable	Median 89 months (4-181)	OS, DFS	★★★	★	★★★	7
Gujam et al., 2014 (35)	UK	361	CD68+	Tertiles	Tumor stroma	Blind	Median 168 months	OS, BCSS	★★★★	★	★★★	8
Yang et al., 2015 (36)	China	100	CD68+	Median 61.14	Tumor nest	Unavailable	Mean 56.68 months	OS	★★★	★	★★	6
Sousa et al., 2015 (37)	Finland	562	CD68+ CD163+	Median CD68: 369 CD163: 167.5	Tumor nest	Double- blinded	Unavailable	DFS	★★★★	★	★★★	8
Gwak et al., 2015 (38)	Korea	276	CD68+	Median 24.2	Tumor nest	Unavailable	Median 7.7 years (0.1-10.6)	DFS	★★★	★★	★★	7
Tiainen et al. 2015 (17)	Finland	270	CD68+ CD163+	Median CD68: 34 CD163: 26	Tumor stroma	Blind	Median 6.3 years (0.4-11.1)	OS	★★★	★★	★★★	8
Ward et al., 2015 (39)	UK	129	CD68+	Mean value	Tumor nest	Unavailable	Median 78 months	DFS	★★★	★	★★	6
Koru-Sengul et al., 2016 (40)	American	150	CD163+	150	Tumor stroma	Blind	Unavailable	OS, DFS	★★★★	★	★★★	8
Tian et al., 2016 (41)	China	278	CD163+	Median 50%	Tumor stroma	Unavailable	Median 76 months (4-116)	OS	★★★	★	★★	6
Shiota et al., 2016 (42)	Japan	167	CD68+	Median 50%	Tumor nest	Blind	Median 86 months (1-159)	OS, BCSS, DFS	★★★★	★	★★★	8
Xu et al., 2017 (43)	China	102	CD68+	Mean number	Tumor stroma	Blind	Unavailable	OS, DFS	★★★★	★	★★★	8
Miyasato et al., 2017 (44)	Japan	149	CD68+ CD163+	190	Tumor nest	Blind	Unavailable	OS, BCSS, DFS	★★★★	★	★★★	8
Liu et al. 2017 (45)	China	203	CD163+	10%	Tumor stroma	Unavailable	Median 51 months (13-88)	OS, DFS	★★★	★★	★★	7
Yang et al. 2018 (46)	China	200	CD68+ CD163+	TN: 11; TS: 36	Tumor nest and stroma	Blind	Median 66 months (12-86)	OS, DFS	★★★	★★	★★★	8
Zhang et al., 2018 (47)	China	278	CD163+	Mean	Tumor nest	Blind	Median 87 months (8-130)	DFS	★★★	★★	★★	7
Yuan et al., 2019 (48)	China	217	CD68+	Immunoreactivity scoring > 6	Tumor nest	Blind	5 years	DFS	★★★	★	★★★	7
Jeong et al., 2019 (49)	Korea	367	CD68+ CD163+	CD68+ TN:33 TS:17.8 CD163+ TN: 1.67 TS: 21	Tumor nest and stroma	Blind	Unavailable	OS, DFS	★★★	★	★★★	7
Jamiyan et al. 2020 (50)	Japan	107	CD68+ CD163+	Median value CD68+ TS: 26.2 TN: 11.2 CD163+ TS: 26.6 TN: 8.6	Tumor nest and stroma	Unavailable	Unavailable	OS, DFS	★★★	★	★★	6
Chen et al., 2020 (51)	Singapore	198	CD68+ CD163+	≥ 10%	Tumor stroma	Unavailable	Median 7.2 years (0-20.4)	DFS	★★★	★	★★★	7
Gunnarsdottir et al., 2020 (52)	Sweden	286	CD68+	10%	Tumor nest	Blind	Unavailable	OS	★★★	★★	★★	7
Lin et al., 2021 (53)	Germany	298	CD68+	≤ 4.5	Tumor stroma	Unavailable	12 years	OS, DFS	★★★	★★	★	6

TN, tumor nest; TS, tumor stroma; OS, overall survival; DFS, disease-free survival; BCSS, breast cancer specific survival; NOS: Newcastle-Ottawa Scale checklist ★: A star means that the study obtain one score in NOS.

For TAM identification, 28 studies used CD68 and 12 studies used CD163, among which three studies used a combination of CD68 and PCNA. Five studies explored the role of TAMs in both TN and TS, 18 studies only detected TAMs in TN, and nine studies only included TAMs in TS. The majority of studies used the median number of macrophages per high-power field as the cut-off value to divide TAMs into the high and low TAM groups. Moreover, most studies assessed the association between TAMs and the prognosis of BCa patients, including OS (25 studies), DFS (24 studies), and BCSS (seven studies). The reported follow-up time ranged from 0.1 to 20.4 years. The NOS scores of all included studies ranged from 6 to 8 (**Table 1**).

### Prognostic Significance of CD68+ TAMs

**Figure 2:**
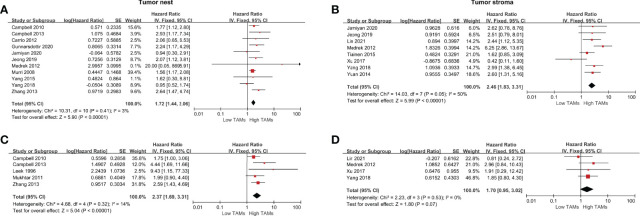
Forest plots of HRs for OS between high and low CD68+ TAM density in BCa patients. **(A)** HRs of OS in raw data for CD68+ TAMs in the TN of BCa; **(B)** HRs of OS in raw data for CD68+ TAMs in the TS of BCa; **(C)** HRs of OS with adjusted measures for CD68+ TAMs in the TN of BCa; **(C)** HRs of OS with adjusted measures for CD68+ TAMs in the TS of BCa.

A total of 15 studies were included in the analysis of CD68+ TAMs on survival data in patients with BCa using the fixed-effect model for the absence of heterogeneity (all *I^2^* <50% or *P*>0.10). Our meta-analysis indicated that a high CD68+ TAM density was significantly associated with poor OS compared to a low CD68+ TAM density in the TN with a pooled HR of 1.72 (95% CI 1.44–2.06, *P*<0.001) and in the TS with a pooled HR of 2.46 (95% CI, 1.83–3.31, *P*<0.001) (**Figures 2A, B**). For adjusted measurements of OS from five studies, the results also supported a poor OS in patients with a high CD68+ TAM density in the TN (HR 2.37, 95% CI 1.69–3.31, *P*<0.001) (**Figures 2C, D**). The results were similar for the association between CD68+ TAMs and BCSS in the TN (HR 1.25, 95% CI 1.03–1.52, *P*=0.03) and TS (HR 2.23, 95% CI 1.68–2.96, *P*<0.001) (**Supplementary Figure 1A**). However, there was no significant association between CD68+ TAMs and BCSS in the TN (HR 0.83, 95% CI 0.33–2.08, *P*=0.70) after excluding the study of Mahmoud et al. for high weight (84.9% of total weight), and the study of Murri et al. for high weight (69.3% of total weight in remaining four studies) (**Supplementary Figure 1B**).

**Figure 3:**
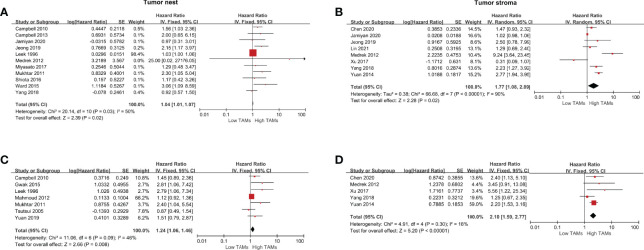
Forest plots of HRs for DFS between high and low CD68+ TAM density in BCa patients. **(A)** HRs of DFS in raw data for CD68+ TAMs in the TN of BCa; **(B)** HRs of DFS in raw data for CD68+ TAMs in the TS of BCa; **(C)** HRs of DFS with adjusted measures for CD68+ TAMs in the TN of BCa; **(D)** HRs of DFS with adjusted measures for CD68+ TAMs in the TS of BCa.

A total of 14 studies were eligible to assess the correlation between CD68+ TAMs and DFS. The results showed that a high CD68+ TAM density in the TS was significantly correlated with shorter DFS compared to a low CD68+ TAMs density (HR 1.77, 95% CI 1.08–2.89, *P*=0.02) in a random-effects model with significant heterogeneity (*I^2^* =90%, *P*<0.001). No significant difference was found in the TN (HR 1.04, 95% CI 1.01–1.07, *P*=0.02) (**Figures 3A, B**). However, the results showed that a high CD68+ TAM density in the TN was significantly correlated with shorter DFS (HR 1.50, 95% CI 1.19–1.89, *P*<0.001) after excluding the study of Leek et al. accounting for 98.4% of total weight (**Supplementary Figure 1C**). For adjusted measurements of DFS from 12 studies, the results support a poor DFS in patients with a high CD68+ TAM density (TN: HR 1.24, 95% CI 1.06–1.46, *P*=0.008; TS: HR 2.10, 95% CI 1.59–2.77, *P*<0.001) (**Figures 3C, D**), and the results still support a poor DFS in patients with a high CD68+ TAM density (TN: HR 1.52, 95% CI 1.16–2.01, *P*=0.003; TS: HR 1.96, 95% CI 1.27–3.02, *P*=0.003) even after excluding the studies of Mahmoud et al. and Yuan et al. accounting for 66.2% and 59.0% of the total weight, respectively (**Supplementary Figure 1D, E**).

### Prognostic Significance of CD163+ TAMs

**Figure 4:**
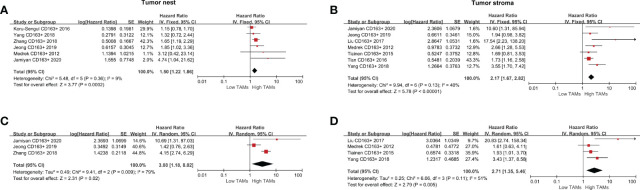
Forest plots of HRs for OS between high and low CD163+ TAM density in BCa patients. **(A)** HRs of OS in raw data for CD163+ TAMs in the TN of BCa; **(B)** HRs of OS in raw data for CD163+ TAMs in the TS of BCa; **(C)** HRs of OS with adjusted measures for CD163+ TAMs in the TN of BCa; **(D)** HRs of OS with adjusted measures for CD163+ TAMs in the TS of BCa.

The following meta-analysis was conducted using the fixed-effect model for the absence of heterogeneity (all *I^2^* <50% or *P*>0.10), except for adjusted measurements of OS in the TN (*I^2 =^* 79%, *P*=0.009). A total of nine studies were eligible to assess the association of CD163+ TAMs and survival data in patients with BCa. The results showed that a high CD163+ TAM density in the TN was significantly associated with poor OS (HR 1.50, 95% CI, 1.22–1.86, *P*<0.001), especially in the TS with a pooled HR of 2.17 (95% CI, 1.67–2.82, *P*<0.001) (**Figures 4A, B**). For adjusted measurements of OS from seven studies, the results also support a poor OS in patients with a high CD68+ TAM density (TN: HR 3.08, 95% CI 1.18–8.02, *P*=0.02; TS: HR 2.71, 95% CI 1.35–5.46, *P*=0.005) (**Figures 4C, D**). There was no significant association between CD163+ TAMs and BCSS in the TN (HR 1.17, 95% CI 0.45–3.05, *P*=0.74), but only two studies were included in this analysis (**Supplementary Figure 1F**).

**Figure 5:**
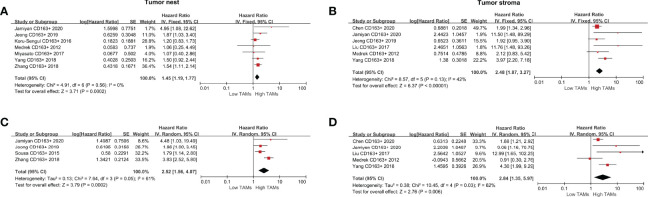
Forest plots of HRs for DFS between high and low CD163+ TAM density in BCa patients. **(A)** HRs of DFS in raw data for CD163+ TAMs in the TN of BCa; **(B)** HRs of DFS in raw data for CD163+ TAMs in the TS of BCa; **(C)** HRs of DFS with adjusted measures for CD163+ TAMs in the TN of BCa; **(D)** HRs of DFS with adjusted measures for CD163+ TAMs in the TS of BCa.

For the correlation between CD163+ TAMs and DFS, the results indicated that a high CD163+ TAM density was significantly associated with shorter DFS both in the TN (HR 1.45, 95% CI 1.19–1.77, *P*<0.001) and TS (HR 2.48, 95% CI 1.87–3.27, *P*<0.001) (**Figures 5A, B**). For adjusted measurements of DFS from eight studies, the random-effects model was used to obtain HRs and the corresponding 95% CIs because the pooled data exhibited high heterogeneity (TN: *I^2 =^* 61%, *P*=0.05; TS: *I^2^ = *62%, *P*=0.03). The results also supported a poor DFS in patients with a high CD163+ TAM density (TN: HR 2.52, 95% CI 1.56–4.07, *P*<0.001; TS: HR 2.84, 95% CI 1.35–5.97, *P*=0.006) (**Figures 5C, D**).

### Association Between TAMs (CD68+ or CD163+) and Clinicopathological Characteristics

We also analyzed the association between TAMs (CD68+ or CD163+) and clinicopathological characteristics in patients with BCa. The pooled results indicated that a high CD68+ TAM density was not significantly associated with age, lymph node status, histology classification, and PR in the TN or TS (all *P*>0.05) (**Table 2**). However, our meta-analysis using a random-effects model also revealed that a high CD68+ TAM density in the TN was significantly associated with larger tumor size (OR 0.36, 95% CI 0.15–0.85, *P*=0.02), no vascular invasion (OR 0.40, 95% CI 0.28–0.58, *P*<0.001), positive Ki-67 (OR 4.23, 95% CI 1.33–13.48, *P*<0.001), positive ER (OR 2.23, 95% CI 1.19–4.18, *P*=0.01), and negative HER-2 (OR 0.08, 95% CI 0.05–0.14, *P*<0.001), with significant heterogeneity (all *I^2^* > 50%).

**Table 2:** Meta-analysis of high CD68+ TAMs density and clinicopathological features of breast cancer patients.

Clinicopathological features	References	No. of studies	Model	Pooled OR (95% CI)	*P* value	Heterogeneity	
						*I^2^* (%)	*P* value
**Tumor nest**							
Age (< 50 y vs ≥ 50 y)	≥ 50 years	9	Random	0.59 (0.33-1.04)	0.07	93	< 0.001
Tumor size (< 2cm vs ≥ 2cm)	≥ 2cm	9	Random	0.36 (0.15-0.85)	0.02	96	< 0.001
Lymph node status (N0 vs. N1-3)	N1-3	7	Random	0.74 (0.13-1.29)	0.28	90	< 0.001
Histological grade (І, II vs III)	III	13	Random	0.85 (0.46-1.56)	0.60	95	< 0.001
Vascular invasion (yes vs no)	No	3	Random	0.40 (0.28-0.58)	< 0.001	55	0.11
Ki-67 status (positive vs negative)	Negative	4	Random	4.23 (1.33-13.48)	0.01	94	< 0.001
ER status (positive vs negative)	Negative	9	Random	2.23 (1.19-4.18)	0.01	94	< 0.001
PR status (positive vs negative)	Negative	7	Random	1.34 (0.88-2.04)	0.17	78	< 0.001
HER-2 status (positive vs negative)	Negative	8	Random	0.08 (0.05-0.14)	< 0.001	88	< 0.001
**Tumor stroma**							
Age (< 50 y vs ≥ 50 y)	≥ 50 years	5	Random	0.48 (0.13-1.85)	0.29	96	< 0.001
Tumor size (< 2cm vs ≥ 2cm)	≥ 2cm	5	Random	0.59 (0.12-2.94)	0.52	97	< 0.001
Lymph node status (N0 vs. N1-3)	N1-3	3	Random	0.71 (0.21-2.42)	0.59	91	< 0.001
Histological grade (І, II vs III)	III	5	Random	0.32 (0.08-1.35)	0.12	97	< 0.001
Vascular invasion (yes vs no)	No	2	Random	0.08 (0.01-2.16)	0.13	94	< 0.001
Ki-67 status (positive vs negative)	Negative	1	–	0.32 (0.21-0.49)	–	–	–
ER status (positive vs negative)	Negative	3	Random	5.00 (3.68-6.80)	< 0.001	94	< 0.001
PR status (positive vs negative)	Negative	3	Random	1.23 (0.60-2.55)	0.57	80	0.006
HER-2 status (positive vs negative)	Negative	3	Random	0.21 (0.01-6.81)	0.38	99	< 0.001

TAMs, tumor-associated macrophages; OR, odds ratio; CI, confidence interval; ER, oestrogen receptor; PR, progesterone receptor; HER-2, human epidermal growth factor receptor-2.

For the association between high CD163+ TAM density and clinicopathological characteristics, pooled analysis showed a significant correlation between high CD163+ TAMs in the TN and age ≥ 50 years (OR 0.21, 95% CI 0.13–0.34, *P*<0.001, random-effects model), large tumor size (OR 0.34, 95% CI 0.12–1.00, *P*=0.05, random-effects model), no vascular invasion (OR 0.56, 95% CI 0.38–0.82, *P*=0.003, fixed-effects model), and positive ER (OR 3.55, 95% CI 2.58–4.88, *P*<0.001, fixed-effects model) (**Table 3**). However, the results of the TS showed no significant association between high CD163+ TAM density and any clinicopathological characteristics, which could be due to insufficient CD163+ TAM data.

**Table 3:** Meta-analysis of high CD163+ TAMs density and clinicopathological features of breast cancer patients.

Clinicopathological features	References	No. of studies	Model	Pooled OR(95% CI)	*P* value	Heterogeneity	
						*I^2^* (%)	*P* value
**Tumor nest**							
Age (< 50 y vs ≥ 50 y)	≥ 50 years	4	Random	0.21 (0.13-0.34)	< 0.001	65	0.04
Tumor size (< 2cm vs ≥ 2cm)	≥ 2cm	5	Random	0.34 (0.12-1.00)	0.05	95	< 0.001
Lymph node status (N0 vs. N1-3)	N1-3	3	Random	0.94 (0.21-4.13)	0.93	95	< 0.001
Histological grade (І, II vs III)	III	5	Random	0.41 (0.13-1.31)	0.13	95	< 0.001
Vascular invasion (yes vs no)	No	2	Fixed	0.56 (0.38-0.82)	0.003	17	0.27
Ki-67 status (positive vs negative)	Negative	2	Random	4.70 (0.88-25.00)	0.07	93	< 0.001
ER status (positive vs negative)	Negative	2	Fixed	3.55 (2.58-4.88)	< 0.001	51	0.15
PR status (positive vs negative)	Negative	1	–	1.81 (0.92-3.57)	0.09	–	–
HER-2 status (positive vs negative)	Negative	2	Random	0.11 (0.01-0.79)	0.03	94	< 0.001
**Tumor stroma**							
Age (< 50 y vs ≥ 50 y)	≥ 50 years	4	Random	1.71 (0.57-5.08)	0.34	90	< 0.001
Tumor size (< 2cm vs ≥ 2cm)	≥ 2cm	5	Random	0.31 (0.06-1.54)	0.15	96	< 0.001
Lymph node status (N0 vs. N1-3)	N1-3	4	Random	1.98 (0.44-8.96)	0.38	95	< 0.001
Histological grade (І, II vs III)	III	5	Random	0.36 (0.06-2.19)	0.27	97	< 0.001
Vascular invasion (yes vs no)	No	1	–	0.03 (0.01-0.09)	–	–	–
Ki-67 status (positive vs negative)	Negative	1	–	2.52 (1.30-4.85)	–	–	–
ER status (positive vs negative)	Negative	2	Random	2.96 (0.61-14.35)	0.18	91	0.001
PR status (positive vs negative)	Negative	3	Fixed	1.22 (0.87-1.71)	0.26	46	0.16
HER-2 status (positive vs negative)	Negative	3	Random	0.25 (0.02-2.53)	0.24	97	< 0.001

TAMs, tumor-associated macrophages; OR, odds ratio; CI, confidence interval; ER, oestrogen receptor; PR, progesterone receptor; HER-2, human epidermal growth factor receptor-2.

### Heterogeneity

We used meta-regression analysis to quantitatively analyze the source of heterogeneity found in **Figure 4B**. A *P*-value <0.1 could be considered the main source of heterogeneity. The results of univariate analysis showed that region, year, sample size, and cut-off value for high or low TAM density may not be the main sources of heterogeneity between studies (**Table 4**). Multivariate analysis also showed that region, year, sample size, and cut-off value may not be a major source of between-study heterogeneity. Subgroup analysis was also conducted for CD68+ TAM density in the TS associated with DFS. The quantitative data for these subgroups are summarized in **Supplementary Table 3**. Subgroup analysis also showed that region, year, sample size, and cut-off value were not the potential sources of heterogeneity (all *P*>0.05).

**Table 4:** Univariable and multivariable meta-regressions for variables.

Variable	Univariable Meta-Regressions			Multivariable Meta-Regression		
	Standard deviation	*P* value	95%CI	Standard deviation	*P* value	95%CI
Region (Europe/Asian)	0.689	0.269	0.56-8.29	0.960	0.660	0.23-10.02
Year (after 2018/before 2018)	0.624	0.527	0.20-2.29	0.813	0.672	0.14-3.49
Sample size (<200/≥200)	0.620	0.571	0.21-2.37	0.990	0.324	0.05-2.62
Cut-off value (not median/median)	0.724	0.465	0.14-2.44	1.164	0.345	0.03-3.26

### Sensitivity Analysis

Due to the significant heterogeneity of CD68+ TAMs and DFS data, sensitivity analysis was conducted to evaluate the stability of the pooled HRs. After excluding individual studies one by one, the pooled HRs did not substantially change. Similarly, we performed sensitivity analysis for the association between CD163+ TAMs and OS data in the TN. When we removed the article by Jeong et al., we found that high CD163+ TAM density in the TN was associated with better OS with no significant heterogeneity (HR 4.30, 95% CI 2.86–6.47, *P*<0.001, *I^2 =^* 0%, *P*=0.39).

### Publication Bias

We examined potential publication bias using funnel plots when the meta-analysis was conducted with more than five studies. The results showed no significant publication bias for TAMs (CD68+ or CD163+) with OS and DFS (**Supplementary Figures 2****,** **3**).

## Discussion

As the leading cause of death among women, BCa remains a significant global health threat, and new therapeutic strategies are required. TAMs are regarded as a potentially promising target for cancer treatment, and increasing studies have explored the possibility to suppress their tumor-promoting activity (54). Recent ongoing pre-clinical TAM-targeted studies indicated that TAMs are closely associated with poor prognosis and BCa progression (55, 56). Given the discordent conclusions among previous studies, the present meta-analysis was conducted to assess the association between TAMs and BCa prognosis.

This meta-analysis included 32 studies analyzing the prognostic value of TAMs in BCa. A total of 15 studies detected TAMs using a CD68+ biomarker, and 11 and eight of these studies identified TAMs in the TN and TS, respectively. CD163 was used in nine studies to identify TAMs, of which six and seven studies evaluated TAMs in the TN and TS, respectively. We systemically analyzed the association between TAMs (CD68+ or CD163+) and OS and DFS in BCa patients. The present study concluded that a high TAM density in the TME was significantly associated with poor prognostic (OS, and DFS) compared to a low TAM density, irrespective of TAM marker (CD68+ or CD163+, all *P*<0.001). Notably, the pooled results were further strengthened by OS and DFS multivariate analyses showing that a high TAM density was significantly related to poorer outcomes (all *P*<0.05). Compared to TAMs detected in the TN, a high TAMs density detected in the TS seems to show relatively higher prognostic value for BCa patients, validated both for CD68+ and CD163+ TAMs. We also analyzed the association between TAMs and clinicopathological characteristics in BCa patients, which indicated that a high TAM density was closely associated with larger tumor size, no vascular invasion, and positive ER. However, the heterogeneity was very large, requiring further clinical studies with larger sample sizes to validate this conclusion.

The conclusion of the present study is in line with two previous meta-analyses, involving 16 studies (57) and 13 studies (58), respectively. The study by Zhao et al. also showed a worse OS in the TS group compared to the TN group (57). Our findings are consistent with these studies, highlighting the significant prognostic value for TAMs in BCa patients. However, there were contradictory conclusions regarding the prognostic value of CD68 and CD163. Zhao et al. reported that CD68 was a more sensitive prognostic indicator than CD163 in BCa patients, while Ni et al. reported the opposite result. Our results indicated that both CD68+ and CD163+ TAMs were significantly related to poor OS and shorter DFS in both raw and adjusted measures. Compared with previous studies, the present meta-analysis has the advantage of a much larger sample size and more included studies, thus providing more reliable conclusions. Our subgroup analysis for different TAM locations (TN and TS), as well as for raw or adjusted measures, provides more insight into the value of TAM location for BCa prognosis.

Our study also found that a high TAM density in the TS tended to have superior prognostic value for BCa than TAMs in the TN. This finding was not only presented for BCa (50, 59), but also for gastric cancer (15) and oral squamous cell carcinoma (60). TAMs are prone to localize in certain cancer tissues and exhibit different biological behaviors (61). A previous study suggested that different histological locations could induce TAMs to perform distinct functions (62). High TAM density in the TS tended to cause stroma activation and extracellular matrix (ECM) remodeling, *via* interacting with other stromal components including lysyl oxidase, matrix metalloproteinase-9, and type IV collagen (63, 64). Fibroblasts and microvessels are the main supporting components for promoting angiogenesis and tumor metastasis. Activation of ECM remodeling enzymes might limit the function of immune cells and keep them out of the tumor (65). The consequences of these factors can result in tumor enlargement and potentially metastasis. However, these niches may be reshaped by anti-cancer therapy. For instance, immunotherapy increased the number of tertiary lymphoid structures, and anti-angiogenic therapy remodeled perivascular system and stroma niches (66). Moreover, several cytotoxic and targeted therapies have been shown to alter the comprehensive phenotype of tumor macrophages (67; 66) .

Although the present meta-analysis indicated that a high TAM density (both in CD68+ and CD163+) is associated with poor prognosis in patients with Bca, the results still need to be treated with caution. CD68 is a universal macrophage marker, as it stains both M1-like and M2-like TAMs, which exerts opposing effects on carcinogenesis. This may be the reason why CD68 was not an independent risk factor for prognosis in some multivariate analyses (29, 30, 46). CD68 can also be detected on some other non-monocyte cells (e.g. fibroblasts) (68, 69). Therefore, CD68 alone may not be a good marker of TAMs to predict OS. CD163 is a highly specific marker for M2-like macrophages. A previous study suggested that the presence of CD163+ TAMs was significantly associated with less favourable clinicopathological features than CD68+ TAMs (29). It has been found that TAMs tend to polarize to M2 in the TME, and their surface receptors and cytokines secreted are similar to M2-like macrophages (70). As a specific and predominant marker of macrophages in BCa, CD163 could be used as a general marker with prognostic impact alone or immunohistochemical double-staining with CD68 to detect macrophage subpopulations and calculate the ratio of M1/M2.

Furthermore, the subgroup analysis indicated that high TMA density was closely related to BCa patients with larger tumor size, no vascular invasion, or positive ER status. This implies that TAMs density may have prognostic, even therapeutic, value for BCa. A study by Castellaro et al. also reported that TAMs could promote proliferation, migration, invasiveness, and breast tumor growth of ER+ cells *via* rendering these estrogen-dependent breast cancer cells resistant to estrogen withdrawal and tamoxifen treatment (71). Therefore, TAM-targeted therapy may help improve BCa prognosis. Currently, several clinical trials on TAM-targeted therapy have been carried out. Interventions targeting TAMs include macrophages depletion, inhibition of macrophage-derived cytokines, anti-TAMs activation, chimeric antigen receptor macrophage (CAR-M) therapy, TAMs-based immune vaccine, and TAMs nanobiotechnology (70). CCL2, CSF-1, and CSF-1R inhibitors have been shown to effectively lower TAM density in both an animal model and clinical trials. (72–74). Given that M1 macrophages exert cytotoxic effects on cancer cells, another novel strategy could focus on inducing pro-tumor TAMs to an anti-tumor phenotype or M1 phenotype using typical agents such as CD40 agonists, CD47 inhibitors, STAT3 inhibitors, Bruton’s tyrosine kinase (BTK) inhibitors, IL-1Ra inhibitors, and TLR agonists (72, 75, 76), However, despite numerous ongoing clinical and pre-clinical trials on TAM-targeting therapies, a further in-depth understanding of the underlying mechanism of TAMs-related carcinogenesis and the complexity of TAM subsets would be essential to fully realize their therapeutic potential.

There are several important strengths of this meta-analysis. First, the present study was the meta-analysis with the largest sample size, including several recently published papers, and thus the pooled results would be more reliable than previous studies. Second, our meta-analysis included different TAMs locations (TN and TS), which adds new information for the impact of TAM location on BCa survival. Third, our results indicated that a high TAM density is significantly related to poorer outcomes, especially for TAMs in the TS, as a useful prognostic marker. Fourth, given that preoperative adjuvant therapy might disturb TAM density, especially for large tumors, ER positive, and Ki-67 positive patients, the reliability of the results may be compromised. Most included studies excluded patients receiving preoperative neoadjuvant chemotherapy or anti-HER2 therapy, increasing the homogeneity of the study population and strengthening the conclusions.

Several limitations of our meta-analysis should be acknowledged. First, there is currently no consensus on the cut-off values of TAMs in BCa, as previous studies did not set a unified criterion. Most included studies adopted a median value as the cut-off for high/low TAMs. Although there is a concern that the inconsistent cut-off values used in the included studies may potentially introduce bias, the univariate and multivariate meta-regression analysis in the present study both demonstrated that the cut-off value was not the potential sources of heterogeneity, indicating studies using different cut-off value were homogeneous, further strengthening the final conclusions. Future large-scale randomized controlled trials and meta-analyses base on individual patient data are warranted to further elucidate the correlation between TAMs and BCa prognosis. Second, there was significant heterogeneity among the analysis of TAMs and clinicopathological features, even when making a distinction between TAM locations. The heterogeneity might be derived from the different antibodies and dilution applications to detect TAM density. Similarly, the cut-off value of Ki-67 expression (14% or 20%) varied in the included studies, which might have introduced heterogeneity. Third, all included articles were retrospective studies, which may have led to selection bias in the pooled results. Fourth, excessive differences in the range of sample sizes may have increased the weight of the studies with big sample sizes in the pooled results and increased systematical biases. Therefore, future studies with larger sample sizes are required to validate the conclusions of our study.

## Conclusion

In summary, the present systemic review and meta-analysis indicates that an elevated density of CD68+ and CD163+ TAMs is associated with poor OS and shorter DFS in BCa patients. Due to the limitations in our study, further well-designed studies with larger sample sizes are needed to validate our conclusion.

## Data Availability Statement

The raw data supporting the conclusions of this article will be made available by the authors, without undue reservation.

## Author Contributions

CW, YZ, QS, and CL designed the project; CW, YL, QS, and CL performed the literature search and data acquisition; CW and YL performed data extraction; FM, HZ, and XH performed the statistical analyses for heterogeneity investigation; CW, HZ, and YZ supported the writing of the paper. All authors read and approved the final manuscript.

## Funding

This study was funded by Key Projects in the National Science and Technology Pillar Program during the Twelfth Five-year Plan Period (No.2014BAI08B00), Beijing Municipal Science and Technology Project (No. D161100000816005), State Key Laboratory of Medicinal Chemical Biology (NanKai University) (No. 2019014) and LAM China Non-profit Organization Special Fund for LAM of Zhejiang Women and Children’s Foundation (No. LAM001-202205). The funding agencies had no role in the design or conduct of the study.

## Conflict of Interest

The authors declare that the research was conducted in the absence of any commercial or financial relationships that could be construed as a potential conflict of interest.

## Publisher’s Note

All claims expressed in this article are solely those of the authors and do not necessarily represent those of their affiliated organizations, or those of the publisher, the editors and the reviewers. Any product that may be evaluated in this article, or claim that may be made by its manufacturer, is not guaranteed or endorsed by the publisher.

## Supplementary Material

Supplementary Table 1 The search strategy of databases.

Supplementary Table 2 Reference of included studies.

Supplementary Table 3 Subgroup analyses to explore the potential sources of heterogeneity for the impact of CD68+ TAMs density in tumor stroma on DFS.

Supplementary Figure 1 Forest plots of HRs for BCa specific survival or DFS between high and low TAM density in BCa patients. **(A)** HRs of BCSS for CD68+ TAMs in the TN of BCa; **(B)** HRs of BCSS for CD68+ TAMs in the TN of BCa after excluding two studies with high weight; **(C)** HRs of DFS in raw data for CD68+ TAMs in the TN of BCa; **(D)** HRs of DFS with adjusted measures for CD68+ TAMs in the TN of BCa; **(E)** HRs of DFS with adjusted measures for CD68+ TAMs in the TS of BCa. **(F)** HRs of BCSS for CD163+ TAMs in the TN of BCa.

Supplementary Figure 2 Funnel plot of studies with CD68+ TAM density for potential publication bias assessment. **(A)** OS and CD68+ TAMs in the TN; **(B)** DFS and CD68+ TAMs in the TN; **(C)** OS in adjusted measurements and CD68+ TAMs in the TN; **(D)** DFS in adjusted measurements and CD68+ TAMs in the TN; **(E)** BCSS and CD68+ TAMs in the TN; **(F)** OS and CD68+ TAMs in the TS; **(G)** OS in adjusted measurements and CD68+ TAMs in the TS; **(H)** DFS in adjusted measurements and CD68+ TAMs in the TS.

Supplementary Figure 3 Funnel plot of studies with CD163+ TAM density for potential publication bias assessment. **(A)** OS and CD163+ TAMs in the TN; **(B)** DFS and CD163+ TAMs in the TN; **(C)** OS and CD163+ TAMs in the TS; **(D)** OS and CD163+ TAMs in the TS.

## Abbreviations

TAMs, Tumor-associated macrophages; BCa, Breast cancer; OS, Overall survival; PR, Progesterone receptor; ER, Estrogen receptor; HER2, Human epidermal growth factor receptor-2; TME, Tumor microenvironment; IHC, Immunohistochemistry; BCSS, Breast cancer specific survival; DFS, Disease-free survival; NOS, Newcastle–Ottawa Scale; TN, Tumor nest; TS, Tumor stroma; HRs, Hazard ratios; CIs, Confidence interval; KM, Kaplan–Meier; OR, Odds risk.
