# Supplementary material for: The Prognostic and Clinical Value of Tumor-Associated Macrophages in Patients With Breast Cancer: A Systematic Review and Meta-Analysis
Source: Front Oncol. 2022 Jun 30;12:905846. doi: 10.3389/fonc.2022.905846 (PMC9280493; doi:10.3389/fonc.2022.905846)
Supplement: Supplementary Table 1 — The search strategy of databases. [file Table_1.docx]

**Supplementary Table 1 The search strategy of databases**

| Database | Search strategy |
| --- | --- |
| Pubmed | #1 "macrophages"[MeSH Terms] |
|  | #2 "macrophages"[mesh] or "tumor-associated macrophage [Title/Abstract]" or "tumor-infiltrating macrophage [Title/Abstract]" or "TAM [Title/Abstract]" or "TAMs [Title/Abstract]" |
|  | #3 " breast neoplasm"[mesh] |
|  | #4 " breast neoplasm"[mesh] or "neoplasms, breast [Title/Abstract]) " or "neoplasms, breast [Title/Abstract]) " or "neoplasm, breast [Title/Abstract])" or " breast tumors [Title/Abstract])" or " breast tumor [Title/Abstract])" or "tumor, breast [Title/Abstract])" or "tumors, breast [Title/Abstract])" or " breast carcinoma [Title/Abstract])" or "carcinoma, breast [Title/Abstract])" or "carcinomas, breast [Title/Abstract])" or " breast carcinomas [Title/Abstract])" or " breast cancer [Title/Abstract])" or "cancer, breast [Title/Abstract])" or "cancers, breast [Title/Abstract])" or " breast cancers [Title/Abstract])" |
|  | #5 #2 and #4 |
|  | #6 #5 Filters: Publication date to 2022/01/31 |
|  | #7 #6 Filters: Humans |
| Embase | #1 'breast tumor'/exp |
|  | #2 'breast neoplasms':ti, ab, kw OR 'neoplasms, breast OR breast neoplasm':ti, ab, kw OR 'neoplasm, breast':ti, ab, kw OR ' breast tumors':ti, ab, kw OR 'tumor, breast':ti, ab, kw OR 'tumors, breast':ti, ab, kw OR 'breast carcinoma':ti, ab, kw OR 'carcinoma, breast':ti, ab, kw OR 'carcinomas, breast':ti, ab, kw OR 'breast carcinomas':ti, ab, kw OR 'breast cancer':ti, ab, kw OR 'cancer, breast':ti, ab, kw OR 'cancers, breast':ti, ab, kw OR 'breast cancers':ti, ab, kw |
|  | #3 #1 OR #2 |
|  | #4 'macrophage'/exp |
|  | #5 'macrophages':ti, ab, kw OR 'tumor-associated macrophage':ti, ab, kw OR 'tumor-infiltrating macrophage':ti, ab, kw OR 'TAM':ti, ab, kw or 'TAMs':ti, ab, kw |
|  | #6 #4 OR #5 |
|  | #7 #3 AND #6 |
|  | #8 #3 AND #6 AND [<1966-2022]/py |
|  | #9 #8 AND [humans]/lim |
| Web of Science | #1 Topic: [Macrophages] |
|  | #2 "tumor-associated macrophage" OR "tumor-infiltrating macrophage" OR "TAM" or "TAMs" |
|  | #3 #1 or #2 |
|  | #4 Topic: [Breast Neoplasms] |
|  | #5 "Breast Cancer" OR "Cancer, Breast" OR "Breast Cancers" OR "Cancers, Breast" OR "Tumors, Breast" OR "Breast Tumors" OR "Breast Neoplasm" OR "Breast Tumor" OR "Neoplasms, Breast" OR "Tumor, Breast" OR "Neoplasm, Breast" OR "Carcinomas, Breast" OR "Carcinoma, Breast" OR "Breast Carcinoma" OR "Breast Carcinomas" |
|  | #6 #4 OR #5 |
|  | #7 #3 and #6 AND 1985-2022 (Year Published) |
