# Supplementary material for: The Prognostic and Clinical Value of Tumor-Associated Macrophages in Patients With Breast Cancer: A Systematic Review and Meta-Analysis
Source: Front Oncol. 2022 Jun 30;12:905846. doi: 10.3389/fonc.2022.905846 (PMC9280493; doi:10.3389/fonc.2022.905846)
Supplement: Supplementary Table 2 — Reference of included studies. [file Table_2.pdf]

Reference

1. Yang J, Li X, Liu X, Liu Y. The role of tumor-associated macrophages in breast carcinoma invasion and metastasis. Int J Clin Exp Pathol. 2015 Jun 1;8(6):6656-64. PMID: 26261547; PMCID: PMC4525881.

2. Sousa S, Brion R, Lintunen M, Kronqvist P, Sandholm J, Mönkkönen J, Kellokumpu-Lehtinen PL, Lauttia S, Tynninen O, Joensuu H, Heymann D, Määttä JA. Human breast cancer cells educate macrophages toward the M2 activation status. Breast Cancer Res. 2015 Aug 5;17(1):101. doi: 10.1186/s13058-015-0621-0. PMID: 26243145; PMCID: PMC4531540.

3. Gwak JM, Jang MH, Kim DI, Seo AN, Park SY. Prognostic value of tumor-associated macrophages according to histologic locations and hormone receptor status in breast cancer. PLoS One. 2015 Apr 17;10(4): e0125728. doi: 10.1371/journal.pone.0125728 PMID: 25884955; PMCID: PMC4401667.

4. Yuan ZY, Luo RZ, Peng RJ, Wang SS, Xue C. High infiltration of tumor-associated macrophages in triple-negative breast cancer is associated with a higher risk of distant metastasis. Onco Targets Ther. 2014 Aug 21;7:1475-80. doi: 10.2147/OTT.S61838. PMID: 25187727; PMCID: PMC4149399.

5. Zhang Y, Cheng S, Zhang M, Zhen L, Pang D, Zhang Q, Li Z. High-infiltration of tumor-associated macrophages predicts unfavorable clinical outcome for node-negative breast cancer. PLoS One. 2013 Sep 30;8(9):e76147. doi: 10.1371/journal.pone.0076147. PMID: 24098773; PMCID: PMC3786995.

6. Campbell MJ, Wolf D, Mukhtar RA, Tandon V, Yau C, Au A, Baehner F, van't Veer L, Berry D, Esserman LJ. The prognostic implications of macrophages expressing proliferating cell nuclear antigen in breast cancer depend on immune context. PLoS One. 2013 Oct 29;8(10):e79114. doi: 10.1371/journal.pone.0079114. PMID: 24205370; PMCID: PMC3812150.

7. Mohammed ZM, Going JJ, Edwards J, Elsberger B, Doughty JC, McMillan DC. The relationship between components of tumour inflammatory cell infiltrate and clinicopathological factors and survival in patients with primary operable invasive ductal breast cancer. Br J Cancer. 2012 Aug 21;107(5):864-73. doi: 10.1038/bjc.2012.347. Epub 2012 Aug 9. PMID: 22878371; PMCID: PMC3426752.

8. Campbell MJ, Tonlaar NY, Garwood ER, Huo D, Moore DH, Khramtsov AI, Au A, Baehner F,

Chen Y, Malaka DO, Lin A, Adeyanju OO, Li S, Gong C, McGrath M, Olopade OI, Esserman LJ. Proliferating macrophages associated with high grade, hormone receptor negative breast cancer and poor clinical outcome. Breast Cancer Res Treat. 2011 Aug;128(3):703-711. doi: 10.1007/s10549-010-1154-y. Epub 2010 Sep 15. PMID: 20842526; PMCID: PMC4657137.

9. Murri AM, Hilmy M, Bell J, Wilson C, McNicol AM, Lannigan A, Doughty JC, McMillan DC. The relationship between the systemic inflammatory response, tumour proliferative activity, T-lymphocytic and macrophage infiltration, microvessel density and survival in patients with primary operable breast cancer. Br J Cancer. 2008 Oct 7;99(7):1013-9. doi: 10.1038/sj.bjc.6604667. Epub 2008 Sep 16. PMID: 18797461; PMCID: PMC2567062.

10. Koru-Sengul T, Santander AM, Miao F, Sanchez LG, Jorda M, Glück S, Ince TA, Nadji M, Chen Z, Penichet ML, Cleary MP, Torroella-Kouri M. Breast cancers from black women exhibit higher numbers of immunosuppressive macrophages with proliferative activity and of crown-like structures associated with lower survival compared to non-black Latinas and Caucasians. Breast Cancer Res Treat. 2016 Jul;158(1):113-126. doi: 10.1007/s10549-016-3847-3. Epub 2016 Jun 9. PMID: 27283835; PMCID: PMC5129629.

11. Yang M, Li Z, Ren M, Li S, Zhang L, Zhang X, Liu F. Stromal Infiltration of Tumor-Associated Macrophages Conferring Poor Prognosis of Patients with Basal-Like Breast Carcinoma. J Cancer. 2018 Jun 6;9(13):2308-2316. doi: 10.7150/jca.25155. PMID: 30026826; PMCID: PMC6036715.

12. Tian W, Wang L, Yuan L, Duan W, Zhao W, Wang S, Zhang Q. A prognostic risk model for patients with triple negative breast cancer based on stromal natural killer cells, tumor-associated macrophages and growth-arrest specific protein 6. Cancer Sci. 2016 Jul;107(7):882-9. doi: 10.1111/cas.12964. Epub 2016 Jun 14. PMID: 27145494; PMCID: PMC4946705.

13. Lin L, Kuhn C, Ditsch N, Kolben T, Czogalla B, Beyer S, Trillsch F, Schmoeckel E, Mayr D, Mahner S, Jeschke U, Hester A. Breast adipose tissue macrophages (BATMs) have a stronger correlation with breast cancer survival than breast tumor stroma macrophages (BTSMs). Breast Cancer Res. 2021 Apr 13;23(1):45. doi: 10.1186/s13058-021-01422-x. PMID: 33849622; PMCID: PMC8042723.

14. Jamiyan T, Kuroda H, Yamaguchi R, Abe A, Hayashi M. CD68- and CD163-positive tumor-associated macrophages in triple negative cancer of the breast. Virchows Arch. 2020 Dec;477(6):767-775. doi: 10.1007/s00428-020-02855-z. Epub 2020 Jun 30. PMID: 32607685;

PMCID: PMC7683466.
15. Yuan J, He H, Chen C, Wu J, Rao J, Yan H. Combined high expression of CD47 and CD68 is a novel prognostic factor for breast cancer patients. Cancer Cell Int. 2019 Sep 11;19:238. doi: 10.1186/s12935-019-0957-0. PMID: 31528120; PMCID: PMC6737685.
16. Jeong H, Hwang I, Kang SH, Shin HC, Kwon SY. Tumor-Associated Macrophages as Potential Prognostic Biomarkers of Invasive Breast Cancer. J Breast Cancer. 2019 Jan 2;22(1):38-51. doi: 10.4048/jbc.2019.22.e5. PMID: 30941232; PMCID: PMC6438840.
17. Gujam FJ, Edwards J, Mohammed ZM, Going JJ, McMillan DC. The relationship between the tumour stroma percentage, clinicopathological characteristics and outcome in patients with operable ductal breast cancer. Br J Cancer. 2014 Jul 8;111(1):157-65. doi: 10.1038/bjc.2014.279. Epub 2014 May 29. PMID: 24874480; PMCID: PMC4090742.
18. Medrek C, Pontén F, Jirström K, Leandersson K. The presence of tumor associated macrophages in tumor stroma as a prognostic marker for breast cancer patients. BMC Cancer. 2012 Jul 23;12:306. doi: 10.1186/1471-2407-12-306. PMID: 22824040; PMCID: PMC3414782.
19. Tiainen S, Tumelius R, Rilla K, Hämäläinen K, Tammi M, Tammi R, Kosma VM, Oikari S, Auvinen P. High numbers of macrophages, especially M2-like (CD163-positive), correlate with hyaluronan accumulation and poor outcome in breast cancer. Histopathology. 2015 May;66(6):873-83. doi: 10.1111/his.12607. Epub 2015 Jan 15. PMID: 25387851.
20. Leek RD, Lewis CE, Whitehouse R, Greenall M, Clarke J, Harris AL. Association of macrophage infiltration with angiogenesis and prognosis in invasive breast carcinoma. Cancer Res. 1996 Oct 15;56(20):4625-9. PMID: 8840975.
21. Mukhtar RA, Moore AP, Nseyo O, Baehner FL, Au A, Moore DH, Twomey P, Campbell MJ, Esserman LJ. Elevated PCNA+ tumor-associated macrophages in breast cancer are associated with early recurrence and non-Caucasian ethnicity. Breast Cancer Res Treat. 2011 Nov;130(2):635-44. doi: 10.1007/s10549-011-1646-4. Epub 2011 Jun 30. PMID: 21717106.
22. Tsutsui S, Yasuda K, Suzuki K, Tahara K, Higashi H, Era S. Macrophage infiltration and its prognostic implications in breast cancer: the relationship with VEGF expression and microvessel density. Oncol Rep. 2005 Aug;14(2):425-31. PMID: 16012726.
23. Mahmoud SM, Lee AH, Paish EC, Macmillan RD, Ellis IO, Green AR. Tumour-infiltrating macrophages and clinical outcome in breast cancer. J Clin Pathol. 2012 Feb;65(2):159-63. doi:

10.1136/jclinpath-2011-200355. Epub 2011 Nov 2. PMID: 22049225.
24. Chen XY, Thike AA, Md Nasir ND, Koh VCY, Bay BH, Tan PH. Higher density of stromal M2 macrophages in breast ductal carcinoma in situ predicts recurrence. Virchows Arch. 2020 Jun;476(6):825-833. doi: 10.1007/s00428-019-02735-1. Epub 2020 Jan 3. PMID: 31897820.
25. Zhang WJ, Wang XH, Gao ST, Chen C, Xu XY, Sun Q, Zhou ZH, Wu GZ, Yu Q, Xu G, Yao YZ, Guan WX. Tumor-associated macrophages correlate with phenomenon of epithelial-mesenchymal transition and contribute to poor prognosis in triple-negative breast cancer patients. J Surg Res. 2018 Feb;222:93-101. doi: 10.1016/j.jss.2017.09.035. Epub 2017 Nov 1. PMID: 29273380.
26. Xu Y, Lan S, Zheng Q. Prognostic significance of infiltrating immune cell subtypes in invasive ductal carcinoma of the breast. Tumori. 2018 Jun;104(3):196-201. doi: 10.5301/tj.5000624. Epub 2018 May 8. PMID: 28430349.
27. Carrio R, Koru-Sengul T, Miao F, Glück S, Lopez O, Selman Y, Alvarez C, Milikowski C, Gomez C, Jorda M, Nadji M, Torroella-Kouri M. Macrophages as independent prognostic factors in small T1 breast cancers. Oncol Rep. 2013 Jan;29(1):141-8. doi: 10.3892/or.2012.2088. Epub 2012 Oct 17. PMID: 23076599.
28. Shiota T, Miyasato Y, Ohnishi K, Yamamoto-Ibusuki M, Yamamoto Y, Iwase H, Takeya M, Komohara Y. The Clinical Significance of CD169-Positive Lymph Node Macrophage in Patients with Breast Cancer. PLoS One. 2016 Nov 18;11(11):e0166680. doi: 10.1371/journal.pone.0166680. PMID: 27861544; PMCID: PMC5115774.
29. Miyasato Y, Shiota T, Ohnishi K, Pan C, Yano H, Horlad H, Yamamoto Y, Yamamoto-Ibusuki M, Iwase H, Takeya M, Komohara Y. High density of CD204-positive macrophages predicts worse clinical prognosis in patients with breast cancer. Cancer Sci. 2017 Aug;108(8):1693-1700. doi: 10.1111/cas.13287. Epub 2017 Jul 3. PMID: 28574667; PMCID: PMC5543503.
30. Ward R, Sims AH, Lee A, Lo C, Wynne L, Yusuf H, Gregson H, Lisanti MP, Sotgia F, Landberg G, Lamb R. Monocytes and macrophages, implications for breast cancer migration and stem cell-like activity and treatment. Oncotarget. 2015 Jun 10;6(16):14687-99. doi: 10.18632/oncotarget.4189. PMID: 26008983; PMCID: PMC4546497.
31. Liu H, Wang J, Zhang M, Xuan Q, Wang Z, Lian X, Zhang Q. Jagged1 promotes aromatase inhibitor resistance by modulating tumor-associated macrophage differentiation in breast cancer patients. Breast Cancer Res Treat. 2017 Nov;166(1):95-107. doi: 10.1007/s10549-017-4394-2.

Epub 2017 Jul 20. PMID: 28730338.

32. Björk Gunnarsdottir F, Auoja N, Bendahl PO, Rydén L, Fernö M, Leandersson K. Co-localization of CD169^{+} macrophages and cancer cells in lymph node metastases of breast cancer patients is linked to improved prognosis and PDL1 expression. Oncoimmunology. 2020 Nov 22;9(1):1848067. doi: 10.1080/2162402X.2020.1848067. PMID: 33299660; PMCID: PMC7714471.
