# Supplementary material for: The Prognostic and Clinical Value of Tumor-Associated Macrophages in Patients With Breast Cancer: A Systematic Review and Meta-Analysis
Source: Front Oncol. 2022 Jun 30;12:905846. doi: 10.3389/fonc.2022.905846 (PMC9280493; doi:10.3389/fonc.2022.905846)
Supplement: Supplementary Table 3 — Subgroup analyses to explore the potential sources of heterogeneity for the impact of CD68+ TAMs density in tumor stroma on DFS. [file Table_3.docx]

Supplementary Table 3 Subgroup analyses to explore the potential sources of heterogeneity for the impact of CD68+ TAMs density in tumor stroma on DFS

| **Subgroup** | **Study number** | **Hazard risk (95%CI)** | ***I*^2^** | ***P* value** |
| --- | --- | --- | --- | --- |
| **Region** |  |  |  |  |
| Europe | 2 | 3.34 (0.48-23.06) | 92% | 0.43 |
| Asian | 6 | 1.51 (0.90-2.52) | 89% |  |
| **Year** |  |  |  |  |
| Before 2018 | 5 | 1.43 (0.99-2.05) | 68% | 0.59 |
| After 2018 | 3 | 2.15 (0.52-8.95) | 89% |  |
| **Sample** |  |  |  |  |
| < 200 | 4 | 1.49 (0.69-3.22) | 89% | 0.37 |
| ≥ 200 | 4 | 2.18 (1.54-3.11) | 32% |  |
| **Cut-off value** |  |  |  |  |
| Not median | 6 | 1.65 (1.05-2.59) | 68% | 0.61 |
| Median | 2 | 2.92 (0.34-2.89) | 95% |  |

TAMs, tumor-associated macrophages; DFS, disease-free survival; CI, confidence interval.
