# Supplementary figures and images for: The Prognostic and Clinical Value of Tumor-Associated Macrophages in Patients With Breast Cancer: A Systematic Review and Meta-Analysis
Source: Front Oncol. 2022 Jun 30;12:905846. doi: 10.3389/fonc.2022.905846 (PMC9280493; doi:10.3389/fonc.2022.905846)

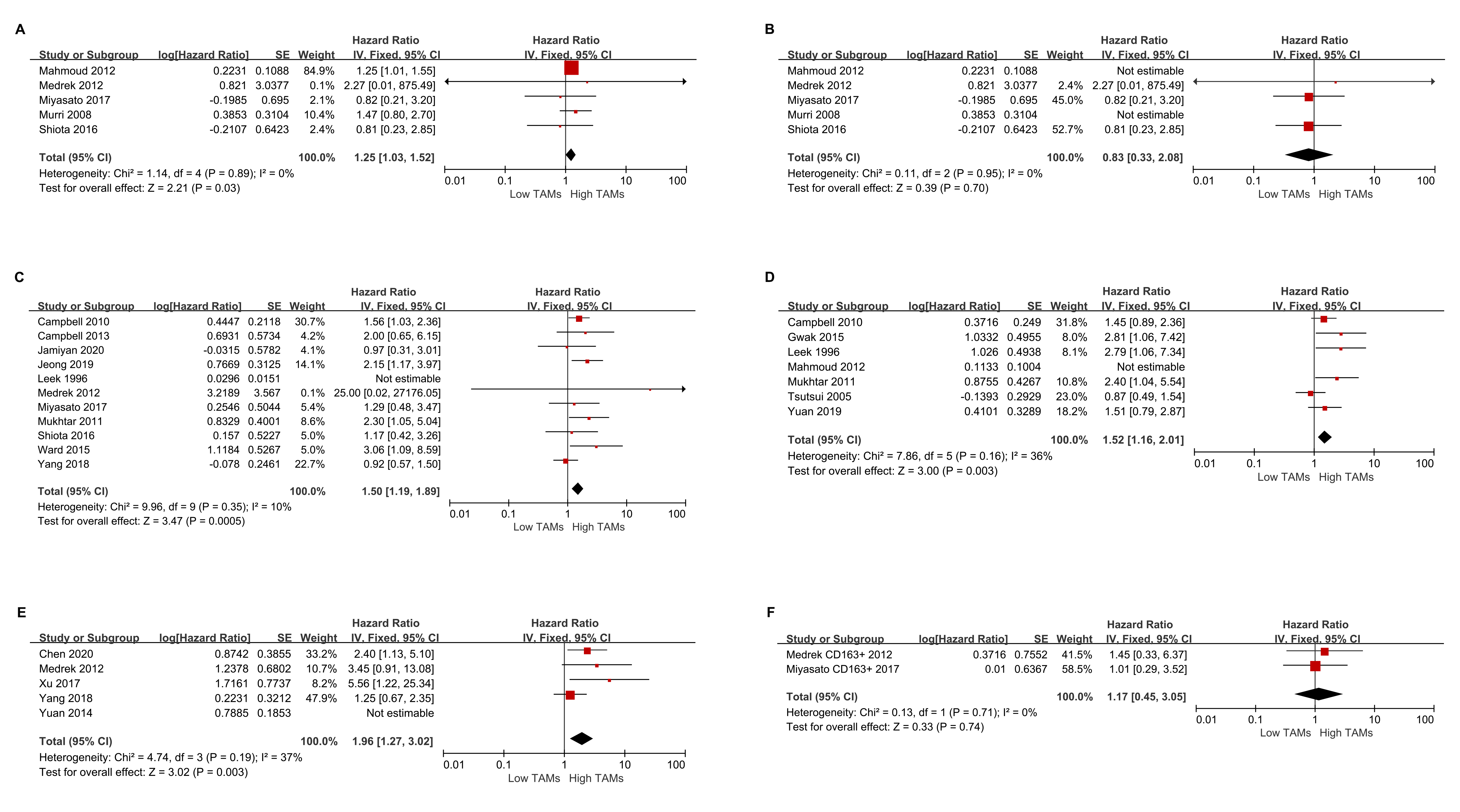

Supplement: Supplementary Figure 1 — Forest plots of HRs for BCa specific survival or DFS between high and low TAM density in BCa patients. (A) HRs of BCSS for CD68+ TAMs in the TN of BCa; (B) HRs of BCSS for CD68+ TAMs in the TN of BCa after excluding two studies with high weight; (C) HRs of DFS in raw data for CD68+ TAMs in the TN of BCa; (D) HRs of DFS with adjusted measures for CD68+ TAMs in the TN of BCa; (E) HRs of DFS with adjusted measures for CD68+ TAMs in the TS of BCa. (F) HRs of BCSS for CD163+ TAMs in the TN of BCa. [file Image_1.tif]

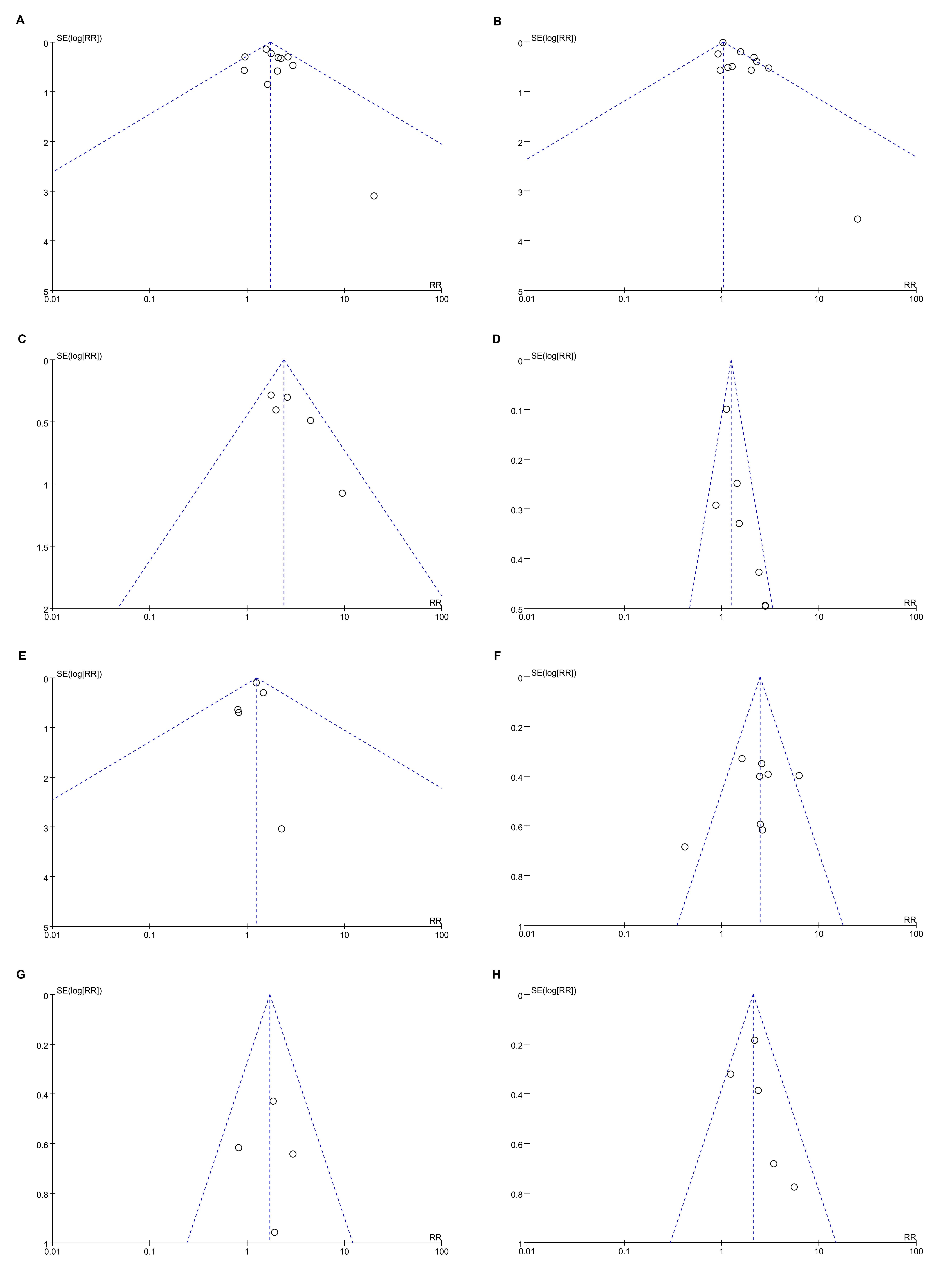

Supplement: Supplementary Figure 2 — Funnel plot of studies with CD68+ TAM density for potential publication bias assessment. (A) OS and CD68+ TAMs in the TN; (B) DFS and CD68+ TAMs in the TN; (C) OS in adjusted measurements and CD68+ TAMs in the TN; (D) DFS in adjusted measurements and CD68+ TAMs in the TN; (E) BCSS and CD68+ TAMs in the TN; (F) OS and CD68+ TAMs in the TS; (G) OS in adjusted measurements and CD68+ TAMs in the TS; (H) DFS in adjusted measurements and CD68+ TAMs in the TS. [file Image_2.tif]

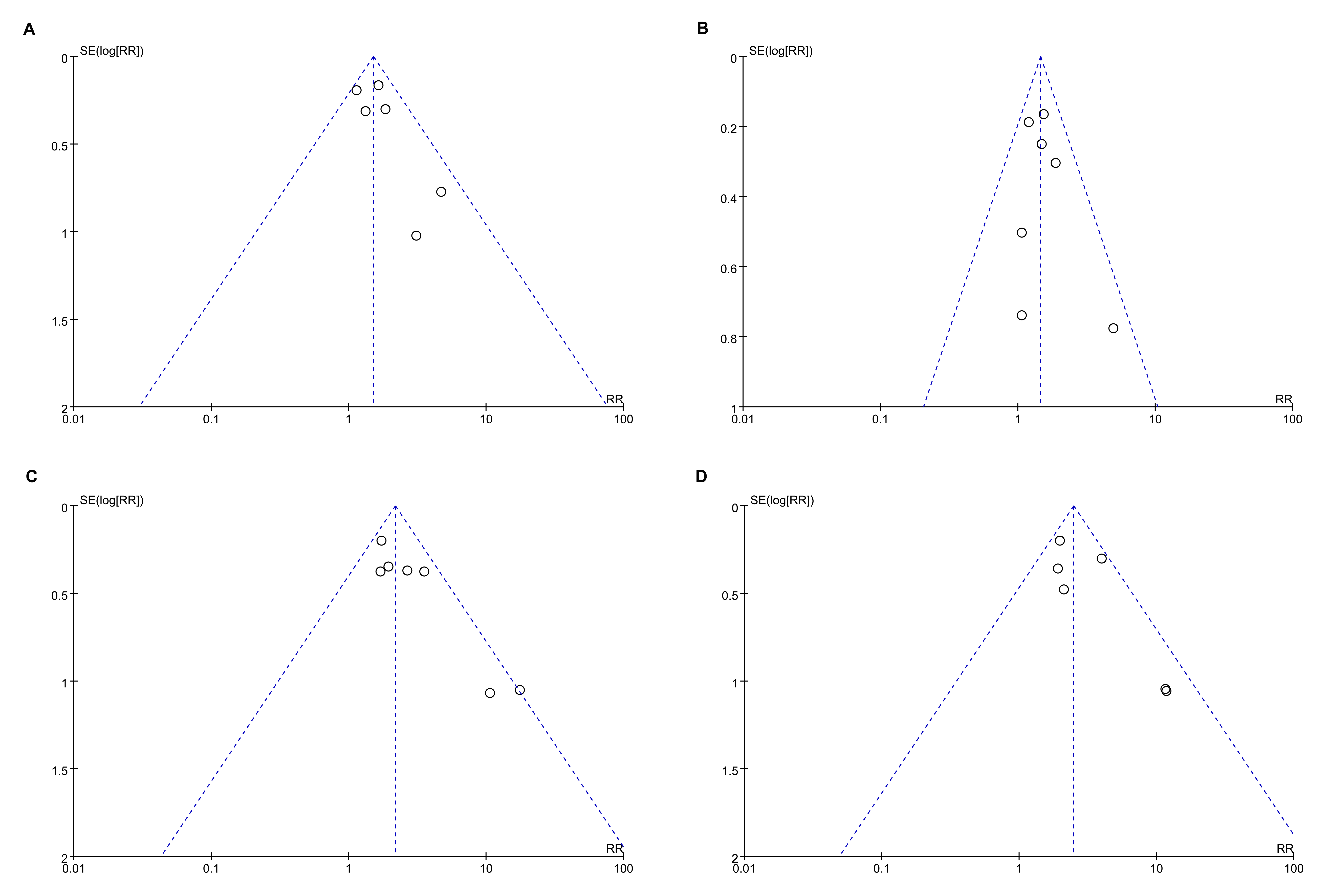

Supplement: Supplementary Figure 3 — Funnel plot of studies with CD163+ TAM density for potential publication bias assessment. (A) OS and CD163+ TAMs in the TN; (B) DFS and CD163+ TAMs in the TN; (C) OS and CD163+ TAMs in the TS; (D) OS and CD163+ TAMs in the TS. [file Image_3.tif]
